# The Impact of Reporting a Prior Penicillin Allergy on the Treatment of Methicillin-Sensitive *Staphylococcus aureus* Bacteremia

**DOI:** 10.1371/journal.pone.0159406

**Published:** 2016-07-20

**Authors:** Kimberly G. Blumenthal, Erica S. Shenoy, Mingshu Huang, James L. Kuhlen, Winston A. Ware, Robert A. Parker, Rochelle P. Walensky

**Affiliations:** 1 Division of Rheumatology, Allergy, and Immunology, Department of Medicine, Massachusetts General Hospital, Boston, Massachusetts, United States of America; 2 Medical Practice Evaluation Center, Department of Medicine, Massachusetts General Hospital, Boston, Massachusetts, United States of America; 3 Harvard Medical School, Boston, Massachusetts, United States of America; 4 Edward P. Lawrence Center for Quality and Safety, Massachusetts General Hospital, Boston, Massachusetts, United States of America; 5 Division of Infectious Disease, Department of Medicine, Massachusetts General Hospital, Boston, Massachusetts, United States of America; 6 Infection Control Unit, Massachusetts General Hospital, Boston, Massachusetts, United States of America; 7 Biostatistics Center, Massachusetts General Hospital, Boston, Massachusetts, United States of America; 8 Acadia Allergy and Immunology, Department of Medicine, University of South Carolina School of Medicine, Greenville, South Carolina, United States of America; Azienda ospedaliero-universitaria di Perugia, ITALY

## Abstract

**Background:**

Methicillin-sensitive *Staphylococcus aureus* (MSSA) bacteremia is a morbid infection with mortality benefit from receipt of parenteral β-lactam therapy. A substantial portion of MSSA bacteremia patients report penicillin allergy, but infrequently have true allergy.

**Objective:**

To determine the frequency and predictors of optimal and adequate therapy in patients with MSSA bacteremia.

**Design:**

Retrospective cohort.

**Participants:**

Adult inpatients with MSSA bacteremia, January 2009 through October 2013.

**Main Measures:**

The primary measure was a trial of optimal therapy (OT), defined as ≥3 inpatient days or discharge on any first-line agents (nafcillin, oxacillin, cefazolin, or penicillin G, if susceptible). The secondary measure was completion of adequate therapy (AT), defined as ≥10 inpatient days or discharge on an agent appropriate for MSSA bacteremia. Data were electronically gathered with key variables manually validated through chart review. Log-binomial regression models were used to determine the frequency and predictors of outcomes.

**Key Results:**

Of 456 patients, 346 (76%) received a trial of OT. Patients reporting penicillin allergy (13%) were less likely to receive OT trial than those without penicillin allergy (47% vs. 80%, p <0.001). Adjusting for other factors, penicillin allergy was the largest negative predictor of OT trial (RR 0.64 [0.49, 0.83]). Infectious Disease (ID) consultation was the largest positive predictor of OT trial across all patients (RR 1.34 [1.14, 1.57]). Allergy/Immunology consultation was the single most important predictor of OT trial among patients reporting penicillin allergy (RR 2.33 [1.44, 3.77]). Of 440 patients, 391 (89%) completed AT, with ID consultation the largest positive predictor of the outcome (RR 1.28 [1.15, 1.43]).

**Conclusions:**

Nearly 25% of patients with MSSA bacteremia did not receive OT trial and about 10% did not receive AT completion. Reported penicillin allergy reduced, and ID consult increased, the likelihood of OT. Allergy evaluation, coupled with ID consultation, may improve outcomes in MSSA bacteremic patients.

## Introduction

*Staphylococcus aureus* (*S*. *aureus*) is the leading cause of community-acquired and hospital-acquired bacteremia [1–5]. Methicillin-sensitive *S*. *aureus* (MSSA) bacteremia comprises a majority of *S*. *aureus* bacteremia and can lead to metastatic infections and death [2,3,6,7]. Treatment of MSSA bacteremia with nafcillin, oxacillin, or cefazolin–parenteral β-lactam antibiotics–has been shown to be associated with less than half the morbidity and mortality when compared with vancomycin, the most commonly used alternative to a β-lactam antibiotic [8–16].

Reported allergy to penicillin antibiotics occurs in 10–15% of hospitalized patients; up to one quarter of MSSA bacteremia cohorts have a reported penicillin allergy [16–18]. However, chart-reported and patient-reported allergies do not equate with true allergy. Among patients who report penicillin allergy who receive allergy evaluation with penicillin skin testing and/or test dose challenges, between 95 to 99% are found not allergic and can tolerate β-lactam antibiotics, including penicillins and cephalosporins (such as cefazolin) [18–24].

While use of intravenous β-lactam therapy is an established quality indicator in MSSA bacteremia, many patients receive suboptimal antibiotic therapy [16,25,26]. Investigators have previously reported on Quality Improvement (QI) interventions in *S*. *aureus* bacteremia, such as mandating specialty consultation of Infectious Diseases (ID) [5,27–30], to improve care. However, prior QI interventions have excluded patients reporting β-lactam allergy or considered vancomycin use appropriate in patients reporting penicillin allergy. These exclusions are despite expert opinion [8,26,31] and simulation-based data [32,33] affirming the importance of addressing penicillin allergy histories when patients present with MSSA [32,33]. We aimed to identify the frequency and predictors of receipt of optimal and adequate therapy among inpatients with MSSA bacteremia, and to assess the impact of reported penicillin allergy on antibiotic treatment.

## Methods

### Study Design and Population

In this retrospective cohort study conducted at the Massachusetts General Hospital (MGH), we examined adult inpatients with an MSSA positive blood culture from January 2009 through October 2013. We excluded patients with a methicillin-resistant *Staphylococcus aureus* blood culture within 30 days of the MSSA culture and patients who died within two days of specimen date (providers would not have known bacterial speciation/sensitivities). For patients with multiple admissions meeting criteria during the study horizon, we considered the index admission.

We used electronic health record (EHR) data to identify patient characteristics, encounter information, allergy history, and antibiotic use. Key data elements for all patients with penicillin allergy and a random 10% sample of patients without penicillin allergy were verified using manual chart review. Discharge antibiotics for all patients were verified by chart review. All chart review was performed by a board-certified internist and allergist/immunologist (KGB or JLK).

This research was approved by the Partners Human Research Committee.

### Setting

MGH is a tertiary care teaching hospital with approximately 48,000 annual admissions located in Boston, Massachusetts. The adult ID service performs consultations when requested by a patient’s primary team. For patients with *S*.*aureus* bacteremia, ID consultation was recommended by the hospital after October 2010. The adult ID service also has an Outpatient Parenteral Antimicrobial Therapy program that monitors MGH inpatients discharged on prolonged parenteral antibiotics to ensure their safe and appropriate treatment. Allergy/Immunology (AI) consultation and penicillin skin testing is available.

### Exposure and Confounding Variables

The primary exposure was a penicillin allergy that was active at the time of the patient’s admission. We defined anaphylaxis, angioedema/swelling, organ involvement (e.g., hepatitis, nephritis), or rashes that involved mucosal lesions or skin desquamation as severe penicillin allergy histories; other penicillin allergy histories were considered not severe. Penicillin allergy histories were additionally categorized as penicillin skin test eligible, based on allergy practice guidelines [18]. Allergies to cephalosporins and vancomycin were identified similarly, and considered as potential confounding variables.

Osteomyelitis, endocarditis, chronic renal failure, and end-stage renal disease (ESRD) were defined using one or more International Classification of Diseases, Ninth Edition (ICD-9) codes billed between the patient’s admission date and discharge date. Consultations were identified by professional billing charges between the admission and discharge date. Length of stay was calculated as the difference between the discharge date and admission date. In-hospital deaths were identified by discharge status, with causes of death identified through chart review.

### Outcome Measures

Antibiotic use was obtained in dose counts of inpatient medication administrations and discharge medications. Optimal therapy antibiotics were parenteral nafcillin, oxacillin, cefazolin, and penicillin; penicillin was only considered optimal if the isolate was penicillin-susceptible [8,26]. Adequate therapy antibiotics included optimal therapy antibiotics as well as other “second-line” parenteral antibiotics, including daptomycin, vancomycin, quinupristin-dalfopristin, linezolid and ceftriaxone [26]. Because of varied literature definitions of adequate MSSA bacteremia coverage [16,27], we additionally used an expanded definition that included telavancin, ceftaroline, carbapenems, ampicillin-sulbactam, piperacillin-tazobactam and ticarcillin-clavulanate. Days of therapy were determined by converting dose counts using standard dosing schedules [34]; when necessary, we adjusted dosing schedules for chronic renal failure and ESRD.

The primary outcome was a trial of optimal therapy, which we defined as ≥3 days of optimal therapy and/or discharge with optimal therapy. The three day duration was chosen because this duration seemed to best capture clinical intent to optimally treat MSSA. We excluded patients who were not alive three days after the MSSA diagnosis since they were ineligible to receive a trial of optimal therapy.

The secondary outcome was adequate therapy completion, defined as discharge with an optimal or adequate therapy; or receipt of ≥10 inpatient days of optimal therapy or adequate therapy. The 10 day duration (rather than 14 days for uncomplicated MSSA bacteremia) [6,31] was chosen because of standard assumptions we made on dosing in the setting of renal insufficiency and the possibility of antibiotic doses not logged in the inpatient administration system (e.g., emergency room, peri-operative, outside hospitals). We excluded patients who were not alive ten days after the MSSA diagnosis since they were not eligible to receive completion of adequate therapy.

For patients who did not receive adequate therapy completion, discharge summaries were reviewed to identify the contributing provider, system and patient factors. Among those with adequate therapy completed, we considered whether each patient was treated with predominantly optimal agents (≥ 10 dosage-days of optimal agents and/or discharged with optimal agents), predominantly “second-line” agents (only used “second-line” agents and/or discharged with “second-line” agents) or a combination of agents (from 1 to 9 dosage-days of optimal agents).

### Impact of Quality Improvement Initiatives

During the study horizon, MGH’s Antibiotic Stewardship Program (ASP) included a note in the electronic microbiologic record reporting *S*. *aureus* that warned that the organism should not be considered a contaminant and recommended ID consultation (implemented October 2010). We evaluated the impact of this note on adequate therapy completion.

A second intervention during the time period was a clinical guideline developed jointly by the MGH ASP and AI for improvement of antibiotic choice among inpatients reporting β-lactam allergy (implemented April 2013) [35,36]. This guideline assisted general inpatient providers in using the beta-lactam allergy history to determine which patients with a history of penicillin allergy could receive beta-lactam antibiotics without additional assessment, which patients could have therapy initiated with an observed challenge dose, and which patients needed penicillin skin testing, obtained only through an AI consultation at MGH. We assessed the impact of this guideline on optimal therapy trial for patients reporting penicillin allergy.

### Statistical Analysis

Data are displayed as frequencies and medians with interquartile ranges. We compared continuous variables using Wilcoxon Rank Sum test and compared frequencies using Fisher’s exact test or Cochrane-Armitage test for trend, as appropriate. We used log-binomial regression models to obtain relative risk estimates for the primary and secondary outcomes including predictors based on *a priori* knowledge or imbalances between groups identified in univariable analyses. We considered p<0.15 as the univariable screening criterion for each potential covariate. Different models were assessed for fit using the Akaike Information Criterion. Our final chosen models included only variables that were significant at p<0.05. We calculated relative risks with 95% confidence intervals. All p-values were 2-sided with p<0.05 considered statistically significant. Statistical analyses were performed in SAS version 9.4 (Cary, NC, USA).

## Results

### Cohort Characteristics

There were 464 unique adult inpatients with MSSA bacteremia from January 2009 through October 2013 at MGH (Table 1). Patients had median age of 60 years [IQR 46 years, 72 years] and 157 (34%) were female. There was little change in the frequency of MSSA bacteremia over calendar time. Renal disease was present in 122 (26%) of patients, with ESRD present in 59 (13%). Allergy to cephalosporins were reported by 20 (4%) and allergy to vancomycin was reported by 18 (4%). Most patients (n = 332, 72%) received an ID consultation. Seventy-one patients (15%) received AI consultation; only 6 (8%) of AI consults occurred in patients who did not also have an ID consult. Patients had a median length of stay of 11 days [IQR 7 days, 18 days], and overall 41 (9%) suffered an in-hospital death. Documented causes of in-hospital deaths included sepsis (n = 20), endocarditis (n = 8), renal failure (n = 3), malignancy (n = 3), aortic dissection/rupture (n = 2), pneumonia (n = 1), myocardial infarction (n = 1), cerebrovascular accident (n = 1), intracranial hemorrhage (n = 1) and gastrointestinal hemorrhage (n = 1).

**Table 1:** Demographic characteristics of inpatients with methicillin-sensitive *Staphylococcus aureus* bacteremia (n = 464).

Characteristic	All (n = 464)	Reported Penicillin Allergy (n = 62)	No Reported Penicillin Allergy (n = 402)	P value*
Age, Median [IQR]	60 [46, 72]	57 [42, 73]	60 [48, 72]	0.53
Female Gender, n (%)	157 (34)	32 (52)	125 (31)	0.002
Year of Hospitalization, n (%)				0.71^†^
*2009*	96 (21)	13 (21)	83 (21)	
*2010*	96 (21)	14 (23)	82 (20)	
*2011*	90 (19)	12 (19)	78 (19)	
*2012*	92 (20)	12 (19)	80 (20)	
*2013*	90 (19)	11 (18)	79 (20)	
Metastatic Infections, n (%)				
*Osteomyelitis*	73 (16)	9 (15)	64 (16)	0.85
*Endocarditis*	128 (28)	16 (26)	112 (28)	0.88
Renal Disease, n (%)	122 (26)	21 (34)	101 (25)	0.13^†^
*Chronic Renal Failure*	63 (14)	10 (16)	53 (13)	
*End Stage Renal Disease*	59 (13)	11 (18)	48 (12)	
Other Drug Allergy History, n (%)				
*Allergy to Cephalosporins*	20 (4)	11 (18)	9 (2)	<0.001
*Allergy to Vancomycin*	18 (4)	5 (8)	13 (3)	0.08
Consultations, n (%)				
*Infectious Diseases*	332 (72)	45 (73)	287 (71)	1.00
*Allergy/Immunology*	71 (15)	16 (26)	55 (14)	0.02
Length of Stay (days), Median [IQR]	11 [7,18]	10 [7, 19]	11 [7, 17]	0.63
In-Hospital Death, n (%)	41 (9)	6 (10)	35 (9)	0.81

* Wilcoxon rank-sum test or Fisher’s exact test, except where specified

^†^ Cochrane-Armitage test for trend

Sixty-two patients (13%) reported prior allergy to penicillin. Patients with reported penicillin allergy were more commonly female (52% vs. 31%, p = 0.002), more likely to have also cephalosporin allergy (18% vs. 2%, p<0.001), and more likely to have AI consultation (26% vs. 14%, p = 0.02). Patients with severe penicillin allergy histories (n = 15, 24%), included patients with anaphylaxis (n = 2), angioedema or swelling (n = 9), shortness of breath (n = 1), hepatitis (n = 1), nephritis (n = 1), and blistering rash (n = 1). The remaining penicillin allergy histories were not severe or would be considered a side effect and/or inconsistent with allergy (n = 47, 76%). Fifty-nine (95%) of reactions were penicillin skin test eligible.

### Primary Outcome, Optimal Therapy Trial

Of 456 patients eligible, 346 (76%) received an optimal therapy trial (Table 2). In univariable analysis, patients with a reported penicillin allergy were less likely to have a trial of optimal therapy than those without a reported penicillin allergy (47% vs. 80%, p<0.001, Table 2). Patients who suffered in-hospital deaths received optimal therapy at a similar frequency than those who did not suffer in-hospital deaths (73% vs 76%, p = 0.67). In multivariable analysis adjusting for year of hospitalization, endocarditis, ESRD, and ID consultation, patients with penicillin allergy were 36% less likely to be given a trial of optimal therapy (RR 0.64 [95% CI 0.49, 0.83], Table 3). The multivariable model also demonstrated that patients with ESRD were also less likely to receive a trial of optimal therapy (RR 0.75 [95% CI 0.60, 0.94]). Patients were more likely to receive an optimal therapy trial if they had an ID consultation (RR 1.34 [95% CI 1.14, 1.57]), endocarditis (RR 1.11 [95% CI 1.03, 1.19]), or hospitalization in a later year (RR 1.04 [95% CI 1.01, 1.07]).

**Table 2:** Antibiotic treatment of inpatients with methicillin-sensitive *Staphylococcus aureus* bacteremia.

	All	Reported Penicillin Allergy	No Reported Penicillin Allergy	P Value*
**Primary Outcome**	(n = 456)	(n = 59)	(n = 397)	
Optimal Therapy Trial, n (%)	346 (76)	28 (47)	318 (80)	<0.001
	All	Reported Penicillin Allergy	No Reported Penicillin Allergy	P Value*
**Secondary Outcome**	(n = 440)	(n = 57)	(n = 383)	
Adequate Therapy Completion, n (%)	391 (89) ^†^	48 (84)	343 (90)	0.26
*Predominantly first-line therapy*	302 (77)	26 (54)	276 (80)	<0.001^‡^
*Predominantly second line therapy*	50 (13)	19 (40)	31 (9)	
*Combination of first-line and second-line therapies*	39 (10)	3 (6)	36 (11)	

* Wilcoxon rank-sum or Fisher’s exact test unless specified

^†^ Of the 49 patients not receiving adequate therapy, 33 were discharged within 10 days without adequate therapy to complete their course, 16 were hospitalized for at least 10 days and not treated with adequate antibiotic therapy for MSSA bacteremia.

^‡^ Cochran-Armitage trend test

**Table 3:** Multivariable log-binomial regression model of optimal therapy trial among inpatients with MSSA bacteremia (n = 456).

	Relative Risk [95% CI]	P Value*
**Factors Associated with *Decreased* Likelihood of Receipt of Optimal Therapy**		
Penicillin Allergy	0.64 [0.49, 0.83]	0.001
End-Stage Renal Disease	0.75 [0.60, 0.94]	0.01
**Factors Associated with *Increased* Likelihood of Receipt of Optimal Therapy**		
Infectious Disease Consultation	1.34 [1.14 1.57]	<0.001
Endocarditis	1.11 [1.03 1.19]	0.004
Later year of hospitalization	1.04 [1.01, 1.07]	0.02

* Wald Chi Square

In univariable analysis among patients reporting penicillin allergy (n = 59), optimal therapy trials were not predicted by age, gender, metastatic infections, renal disease, other drug allergy histories, nor related to hospital length of stay, nor in-hospital death (Table 4). The severity of the reported penicillin reaction was not related to whether or not such patients received a trial of optimal therapy (p = 0.89). ID consultation (86% vs. 58%, p = 0.02), AI consultation (46% vs. 10%, p = 0.003), and later year of hospitalization (p = 0.004) were, however, associated with optimal therapy trials. In the multivariable regression model, AI consultation was the only significant predictor of optimal therapy trial among patients who report prior penicillin allergy (RR 2.33 [1.44–3.77]).

**Table 4:** Univariable analyses assessing predictors of optimal therapy trial in MSSA bacteremia and Reported Penicillin Allergy (n = 59).

	Optimal Therapy Trial(n = 28)	No Optimal Therapy Trial(n = 31)	P value*
**General Characteristics**			
Age, Median [IQR]	57 [41, 72]	54 [44, 74]	0.98
Female Gender, n (%)	14 (50)	17 (55)	0.80
Year of Hospitalization, n (%)			0.004^†^
*2009*	3 (11)	10 (32)	
*2010*	6 (21)	7 (23)	
*2011*	3 (11)	8 (26)	
*2012*	8 (29)	4 (13)	
*2013*	8 (29)	2 (6)	
Metastatic Infections, n (%)			
*Osteomyelitis*	5 (18)	4 (13)	0.72
*Endocarditis*	8 (29)	7 (23)	0.77
Renal Disease, n (%)	8 (29)	11 (35)	0.42^†^
*Chronic Renal Failure*	5 (18)	5 (16)	
*End Stage Renal Disease*	3 (11)	6 (19)	
Other Drug Allergy History, n (%)			
*Allergy to Cephalosporins*	4 (14)	6 (19)	0.73
*Allergy to Vancomycin*	1 (4)	4 (13)	0.36
Consultation Use, n (%)			
*Infectious Disease*	24 (86)	18 (58)	0.02
*Allergy/Immunology*	13 (46) ^‡^	3 (10)	0.003^ǁ^
Length of Stay, Median [IQR]	12 [8, 19]	9 [7, 23]	0.53
In-Hospital Death, n (%)	2 (7)	1 (3)	0.60
**Penicillin Allergy History Characteristics**			
*Severe reaction history*	7 (25)	7 (23)	1.00
*Anaphylaxis*	1 (4)	1 (3)	1.00
*Angioedema*	3 (11)	3 (10)	
*Severe reaction history other than anaphylaxis or angioedema*	3 (11)	3 (10)	

* Wilcoxan Rank Sum test or Fisher’s exact test, unless specified

^†^ Cochrane-Armitage test

^‡^ Included 6 patients (46%) who received observed challenge doses without preceding penicillin skin testing, 5 patients (38%) who received an observed challenge dose after negative penicillin skin testing, and 2 patients (15%) who received their initial dose by a desensitization procedure.

^ǁ^ Adjusted analysis in multivariable model remains significant with RR 2.33 [1.44 3.77]

### Secondary Outcome, Adequate Therapy Completion

Of 440 patients eligible for receipt of adequate therapy completion, 391(89%) of patients with MSSA bacteremia received adequate therapy (Table 2, bottom). Of the 49 patients eligible who did not receive adequate therapy, 33 were discharged within 10 days without adequate therapy to complete their course, and 16 were hospitalized for at least 10 days and not treated with adequate inpatient therapy for MSSA bacteremia. Among the 16 hospitalized for at least 10 days, 3 subsequently suffered in-hospital deaths. We identified patient causes for failure to complete adequate therapy in 6 of these 16, including patient left against medical advice (n = 4), treatment refused by patient (n = 1), and treatment not consistent with goals of care (n = 1).

Among patients completing adequate therapy, a majority (n = 302, 77%) completed therapy with predominantly optimal agents. Fewer patients completed therapy with only alternative agents (n = 50, 13%) and a combination of agents (n = 39, 10%). Although a smaller proportion of patients with a reported penicillin allergy received adequate therapy completion than patients without a penicillin allergy history, this difference was not statistically significant (84% vs. 90%, p = 0.26), but the type of therapy received (optimal, secondary, mixed) was different (p < 0.001). Using the expanded definition of adequate therapy resulted in similar results with 395 (90%) of patients receiving adequate therapy.

In the multivariable regression model of adequate therapy completion, the strongest predictor was ID consultation (RR 1.28 [1.15, 1.43]). Later year of hospitalization (RR 1.02 [1.00, 1.03] was also associated with receipt of adequate therapy.

### Impact of Quality Improvement Initiatives

After the electronic note in the microbiology record, significantly more patients received adequate therapy completion compared to before (92% vs 83%, p = 0.003). After implementation of the standardized guideline for patients with β-lactam allergy, significantly more patients reporting penicillin allergy received a trial of optimal therapy compared to before (88% vs 41%, p = 0.02). In assessing the impact of QI implementation dates in the multivariable models, however, hospitalization year (i.e., step-wise improvement over time) fit better than pre/post these QI interventions.

## Discussion

In this retrospective cohort analysis of adult inpatients with MSSA bacteremia, 9% of patients suffered in-hospital deaths, 24% did not receive a trial of optimal therapy, and 11% did not receive adequate therapy completion. Patients reporting penicillin allergy were 36% less likely to receive a trial of optimal therapy with indicated β-lactam antibiotics. We found no relationship between optimal therapy trial and severity of the reported reaction to penicillin, which suggests that there was no allergy-history-driven logic behind who did—and who did not—receive optimal therapy. Furthermore, a majority of patients (95%) could have had a penicillin skin test if testing had been pursued. Our data confirmed that patients with MSSA bacteremia have improved antibiotic choice and guideline-concordant care when ID consultation is obtained. Last, we identified the importance of allergy evaluation in MSSA bacteremia patients with reported penicillin allergy.

We found that 9% of patients with MSSA bacteremia suffered in-hospital deaths, similar to a previous report that found 6% of patients with MSSA bacteremia suffered an in-hospital death, and consistent with the overall mortality range reported in MSSA bacteremia (9–50%) [3,10,13,14,16]. While we observed that the inpatient burden of this disease is substantial, MSSA infections additionally affect over 80,000 outpatients per year in the US [37–40].

Although MSSA bacteremia leads to untoward outcomes, about 1 in 10 patients did not meet our criteria for being treated adequately, even using an expanded definition of adequate treatment. Patients inadequately treated included patients treated with inappropriate agents (e.g., oral trimethoprim-sulfamethoxazole), treated with appropriate agents for an inappropriately short duration, or not treated at all (e.g., MSSA was considered a contaminant). However, not all inadequate cases reflected failure by medical providers and/or the health system, since patient factors such as goals of care and refusal of treatment additionally contributed. Still, these observations imply that there is room for improvement in the care of patients with MSSA bacteremia.

ID consultation was obtained in 72% of patients overall, and was positively and strongly associated with the patient receiving a trial of optimal therapy and completion of adequate therapy. These data provide additional support for using ID consultation to improve outcomes in MSSA bacteremia [5,8,27–30]. For hospitals that cannot feasibly implement routine ID consultation, a clinical decision support system could leverage ID best practices by requiring a specific order set, including the optimal antibiotic choice and duration of therapy, when a blood culture returns with MSSA.

Only 47% of patients with MSSA with reported penicillin allergy received a trial of optimal therapy. We identified that among the patients reporting penicillin allergy, AI consultation was the strongest predictor for the patient receiving a trial of optimal therapy. This is not surprising because 95% of patients with reported penicillin allergy were skin-test eligible and historically, over 95% of those reporting penicillin allergy can tolerate β-lactam antibiotics [18–24]. Although performing penicillin skin testing would be the ideal approach to inpatients with MSSA infections reporting penicillin allergy, because cefazolin has low (˂3%) cross reactivity with penicillin [41–46], a detailed allergy history would allow for use of cefazolin use directly by some patients reporting penicillin allergy without prior penicillin skin testing. This is important because many hospitals are without access to inpatient AI consultation, and currently, only about 600 US hospitals (<10%) have major determinant (benzylpenicilloyl polylysine or Pre-Pen®), the penicillin skin testing reagent, on formulary [47,48]. However, in addition to allergists, registered nurses, pharmacists, and/or clinicians from other specialties can be trained to perform penicillin skin testing [21,24,35,49].

In retrospective pre/post analysis, we observed a 9% increase in adequate therapy completion resulting from a note in the microbiology record. This was a simple and targeted educational QI initiative, but there was no method to track that the note was read and understood by the clinical team. Future work may link the microbiological record to educational alert and/or an order set. We observed an almost 50% improvement in optimal therapy trial resulting from a standardized guideline for inpatients reporting β-lactam allergies. Because understanding penicillin allergy and cross reactivity with other β-lactam antibiotics is challenging [36,50,51], hospitals may benefit from a similar approach to patients with prior reported β-lactam allergies [35,49,52,53].

This analysis has a number of limitations. First, we used a retrospective study design. However, selection bias was minimized by defining a complete MSSA inpatient cohort through use of the microbiology record, and through identifying high quality objective measures for the primary exposure and outcomes obtained through the electronic health record. Although we attempted to reduce misclassification through manual validation of important variables and use of antibiotic dose counts—instead of team notes or orders, for example—in documenting our primary and secondary outcomes, potential misclassification could still exist. We were limited in our conversion of dosing schedules in renal insufficiency for antibiotics dosed by renal function (e.g., cefazolin, vancomycin). While we needed to choose standard cut-offs for doses per day, renal function is often a moving target among inpatients, and we were unlikely to have captured some important fluctuations associated with acute kidney injury. We therefore chose a somewhat liberal definition of optimal therapy trial (3 days) and adequate therapy completion (10 days), both of which were set *a priori* to data analysis. We did not account for all possible confounders of antibiotic choice, including all underlying illnesses, physical fragility, support at home, and dosing convenience (e.g., beta-lactams are often dosed more frequently than vancomycin). Fortunately, many of these factors should not be related to the primary exposure (penicillin allergy), and would therefore be unlikely to yield different results. We were unable to examine changes in practice by medical unit or patient acuity, which could be useful in identifying opportunities for targeted interventions. Finally, our results represent the experience of only one academic medical center, although MGH has similar patient populations and resources as many other tertiary care centers in the US and internationally.

This large, single-center retrospective cohort analysis of patients with MSSA bacteremia demonstrates that there is considerable room for improvement in the care of all patients with MSSA bacteremia, especially those who report prior penicillin allergy. Because clinical outcomes, including less recurrence, metastases, and death, are fewer with delivery of optimal therapy, efforts may be best focused on encouraging or mandating ID consultation or incorporation of ID guidelines into EHR decision support. Improving the inpatient penicillin allergy assessment will help ensure the best treatment for patients with reported penicillin allergy.
